# Is high-frequency oscillatory ventilation more effective and safer than conventional protective ventilation in adult acute respiratory distress syndrome patients? A meta-analysis of randomized controlled trials

**DOI:** 10.1186/cc13900

**Published:** 2014-05-30

**Authors:** Xiao-ling Gu, Guan-nan Wu, Yan-wen Yao, Dong-hong Shi, Yong Song

**Affiliations:** 1 Department of Respiratory Medicine, Jinling Hospital, Nanjing University School of Medicine, 305 East Zhongshan Road, Nanjing, Jiangsu Province 210002, P. R. China; 2 Department of Medical Imaging, Jinling Hospital, Nanjing University School of Medicine, 305 East Zhongshan Road, Nanjing, Jiangsu Province 210002, P. R. China

## Abstract

**Introduction:**

Comprehensively evaluating the efficacy and safety of high-frequency oscillatory ventilation (HFOV) is important to allow clinicians who are using or considering this intervention to make appropriate decisions.

**Methods:**

To find randomized controlled trials (RCTs) comparing HFOV with conventional mechanical ventilation (CMV) as an initial treatment for adult ARDS patients, we searched electronic databases (including PubMed, MedLine, Springer Link, Elsevier Science Direct, ISI web of knowledge, and EMBASE) with the following terms: “acute respiratory distress syndrome”, “acute lung injury”, and “high frequency oscillation ventilation”. Additional sources included reference lists from the identified primary studies and relevant meta-analyses. Two investigators independently screened articles and extracted data. Meta-analysis was conducted using random-effects models.

**Results:**

We included 6 RCTs with a total of 1,608 patients in this meta-analysis. Compared with CMV, HFOV did not significantly reduce the mortality at 30 or 28 days. The pooled relative risk (RR) was 1.051 (95% confidence interval (CI) 0.813 to 1.358). ICU mortality was also not significantly reduced in HFOV group, with a pooled RR of 1.218 (95% CI 0.925 to 1.604). The pooled effect sizes of HFOV for oxygenation failure, ventilation failure and duration of mechanical ventilation were 0.557 (95% CI 0.351 to 0.884), 0.892 (95% CI 0.435 to 1.829) and 0.079 (95% CI −0.045 to 0.203), respectively. The risk of barotrauma and hypotension were similar between the CMV group and HFOV group, with a RR of 1.205 (95% CI 0.834 to 1.742) and a RR of 1.326 (95% CI 0.271 to 6.476), respectively.

**Conclusions:**

Although HFOV seems not to increase the risk of barotrauma or hypotension, and reduces the risk of oxygenation failure, it does not improve survival in adult acute respiratory distress syndrome patients.

## Introduction

Both acute lung injury (ALI) and acute respiratory distress syndrome (ARDS) are life-threatening conditions that are usually associated with substantial morbidity [1,2], mortality [3], and financial costs [4]. Conventional mechanical ventilation (CMV) is still considered the cornerstone of treatment for these patients. However, although mechanical ventilation can initially sustain life, it may cause further lung injury [5-8].

To avoid ventilator-induced lung injury, lung-protective ventilation has been recommended, which focused on avoiding cyclic alveolar collapse and re-expansion, preventing alveolar excess distension, and achieving and maintaining alveolar recruitment [9-11]. High-frequency oscillation is an alternative mechanical ventilation method that delivers very small tidal volumes at high frequencies (3 to 15 Hz) using an oscillatory pump [12]. High-frequency oscillatory ventilation (HFOV) can not only avoid over-distension of alveoli by delivering small tidal volumes but can also prevent end-expiratory alveolar collapse and maintain alveolar recruitment by applying a constant airway pressure [13-15]. Therefore, HFOV theoretically achieves all goals pursued by lung-protective ventilation strategies [11,16,17].

However, no more than six randomized controlled trials (RCTs) in adult ARDS patients have been published on the safety and efficacy of HFOV as an initial treatment strategy. Three previous trials comparing HFOV with CMV suggested that HFOV improved both oxygenation and survival in adults with ARDS [18-20], but two recent larger-scale RCTs presented different or even opposite results [21,22]. Therefore, this approach remains an unproven and controversial therapy for adults with ARDS [23-26].

Two Cochrane reviews examining the effect of HFOV on mortality in ALI/ARDS patients have been published. The earlier one found only two small RCTs and was not powerful enough to draw definitive conclusions [27]; the later study [28] concluded that HFOV might improve survival, which was not completely consistent with the conclusions of two recently published large RCTs [21,22]. Neither of the above two Cochrane reviews focused on the effect of HFOV in unique adults with ARDS. Since two large scale RCTs comparing HFOV with CMV as an initial treatment for unique adult ARDS patients have been recently published, we performed a meta-analysis of RCTs, to systematically review the efficacy and safety of HFOV in the population of adult ARDS patient compared with CMV.

## Methods and materials

### Ethics statement

We performed a meta-analysis of published RCTs comparing HFOV with CMV for ALI/ARDS in unique adult patients. All analyses were based on published data extracted from the six eligible studies, which have been approved by the Institutional Review Committee on Human Research. An additional file shows this in more detail (see Additional file 1). Additionally, as described in the six primary studies, all patients (or their representatives) enrolled in these six trials have provided written informed consent before any study-related procedure was performed. Therefore, the present meta-analysis does not present any further problems in relation to ethics or conflicts of interest.

### Literature search and identification of the publications

To identify all published RCTs comparing HFOV with CMV in adult ARDS patients, a search of electronic databases (including PubMed, MedLine, Springer Link, Elsevier Science Direct, ISI web of knowledge, and EMBASE) was carried out with the following terms: acute respiratory distress syndrome; acute lung injury; high frequency oscillation ventilation. There were no language restrictions. This search was conducted through July 2013, with no additional time limits. The reference lists of the identified primary studies and relevant meta-analyses were also searched for additional studies.

To be included in the present meta-analysis, studies had to be RCTs comparing HFOV with CMV, enrolling unique adult ALI/ARDS patients, and reporting at least one of the following outcomes of interest: ICU mortality; 28- or 30-day mortality; hypoxemia and ventilation failure; duration of mechanical ventilation; and the incidence of barotrauma or hypotension. All of the candidate articles were independently read and checked for the inclusion criteria by two investigators (XG and GW). Disagreements were resolved through consensus. The methodological quality of the included studies was evaluated according to the Cochrane handbook 5.1.0 for randomized controlled trials [29]. Given that blinding of physicians, patients or related family members was impossible in those trials, we compared whether rescue treatments were equally applied in the treatment group and control group, and performed quality assessment according to the following five aspects, including random sequence generation, allocation concealment, incomplete outcome data, selective reporting, and others.

### Data extraction

Information was extracted independently by two investigators (XG and GW) from all eligible studies. The required items included in the data form were as follows: (1) basic information about the primary study, including the first author’s name, year of publication, sample size of the study, single or multicenter design, the definition of ALI or ARDS used, and the overall risk of bias; (2) clinically relevant primary outcomes, including ICU mortality, 28- or 30-day mortality, hypoxemia (including oxygenation failure and refractory hypoxemia diagnosed in the primary eligible studies) and ventilation failure (including ventilation failure, acidosis, and refractory acidosis diagnosed in the primary eligible studies), and duration of mechanical ventilation; and (3) the incidence of barotrauma or hypotension. The lists from the two investigators were compared, and disagreements about the extracted data were resolved by consensus.

### Statistical analyses

All meta-analysis were performed by random-effects models (the DerSimoniane and Laird method). We reported continuous outcomes using standardized mean differences with the 95% CI, and binary outcomes were presented as relative risk with the 95% CI. The *Z*-test and chi-square test were used to generate the *P*-value for the continuous outcomes and for the binary outcomes, respectively. *P* <0.05 was considered statistically significant.

The presence of heterogeneity between studies was tested with the chi-square-based *Q*-test and quantified with the *I*^2^ statistic (25 to 49% for low heterogeneity, 50 to 74% for moderate heterogeneity, and 75 to 100% for high heterogeneity) [30]. Sensitivity analysis was performed to evaluate the influence of the individual trial on the pooled effect. Potential publication bias was investigated by funnel plots and was formally evaluated with Egger’s linear regression test and Begg’s adjusted rank correlation test. All statistical analyses were performed with Stata software (version 11.0; StataCorp LP, College Station, TX, USA) using two-sided p values. *P* <0.05 was considered statistically significant.

## Results

### Literature search and study characteristics

**Figure 1:**
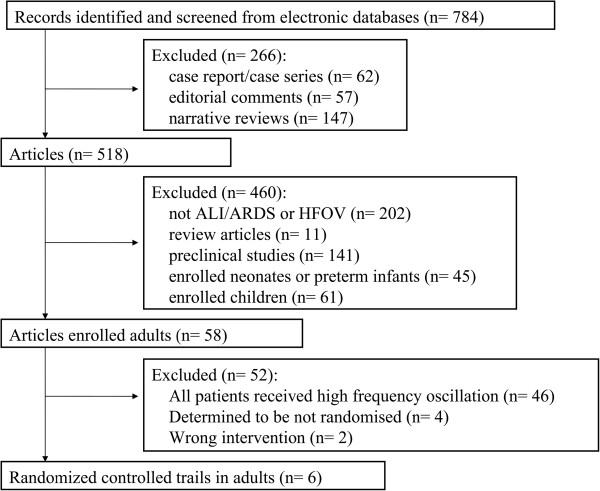
Flow chart of studies included in this meta-analysis.

Using the search term *high frequency oscillatory ventilation* combined with *acute respiratory distress syndrome* or with *acute lung injury*, 784 citations were identified. Among them, six trials [18-22,31] met the inclusion criteria and were enrolled in the meta-analysis. The flow chart of the identification and selection of publications is shown in Figure 1.

Six eligible trials [18-22,31] enrolled a total of 1,608 adult patients with ARDS (Table 1). All trials investigated HFOV as an initial treatment for ARDS rather than as a rescue treatment after the failure of conventional ventilation. The tested patients in most of these trials were continuously treated with HFOV for more than 24 hours, except in one trial that continuously applied high-frequency oscillation for 12 hours [20]. The control groups in four trials underwent low-tidal-volume ventilation (≤8 ml/kg) [20-22,31], whereas three trials performed low plateau pressure (≤35 cm H_2_O) [20,21,31]. Among all of the included studies, five studies with high methodological quality and low risk of bias passed the quality assessment [18,20-22,31], and the risk of bias in the sixth trial was unclear [19] (Table 1).

**Table 1:** Essential characteristics of included studies

**First author**	**Year**	**Institute**	**Patients (number)**		**Details of ARDS**	**Overall risk of bias**
			**HFOV**	**CMV**		
Derdak [18]	2002	ICUs in 13 US hospitals	75	73	ARDS; PEEP <10 cm H_2_O	Low
Shah [31]	2004	1 ICU in Cardiff, Wales	15	13	ARDS	Low
Bollen [19]	2005	5 ICUs in 4 European cities	37	24	ARDS	Unclear (>10% (11/61) crossovers)
Demory [20]	2007	1 ICU in Marseille, France	13	15	ARDS; PaO_2_/FiO_2_ ≤ 150, PEEP ≥5cmH2O	Low
Young [22]	2013	ICUs in England, Wales, and Scotland	398	397	ARDS; PaO_2_/FiO_2_ ≤ 200, PEEP ≥5cmH2O	Low
Ferguson [21]	2013	38 centers in Canada, the United States, Saudi Arabia, Chile, and India	275	273	ARDS; PaO_2_/FiO_2_ ≤ 200,FiO_2_ ≥ 0.5	Low

ARDS, acute respiratory distress syndrome; CMV, conventional mechanical ventilation; FiO_2_, fraction of inspired oxygen; HFOV, high-frequency oscillatory ventilation; PaO_2_, arterial oxygen tension; PEEP, positive end expiratory pressure.

### Mortality at 28 or 30 days

**Figure 2:**
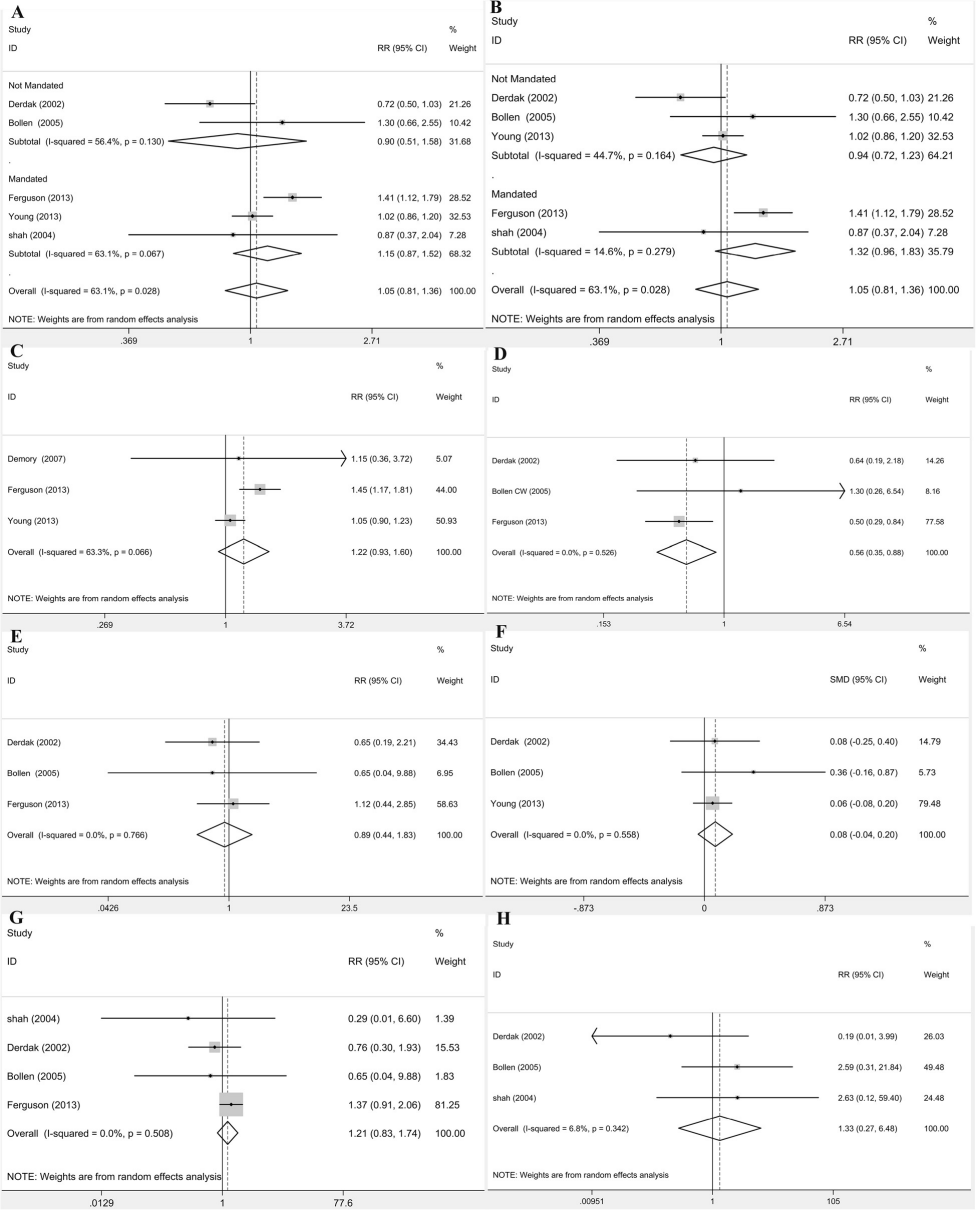
**Forest plots for the meta-analysis of mortality: (A) Meta-analysis of mortality at 28 or 30 days, and subgroup analyses stratified by the tidal volume of the conventional ventilation group (in which tidal volumes ≤8 ml/kg were used or not used). (B)** Meta-analysis of mortality at 28 or 30 days, and subgroup analyses stratified by the plateau pressure of the conventional ventilation group (in which plateau pressure ≤35 cmH_2_O was used or not used). **(C)** Meta-analysis of ICU mortality. **(D)** Meta-analysis for oxygenation failure. **(E)** Meta-analysis of ventilation failure. **(F)** Meta-analysis for the duration of mechanical ventilation. **(G)** Meta-analysis of barotrauma. **(H)** Meta-analysis of hypotension. LPV, lung-protective ventilations.

In the primary analysis of five trials [18,19,21,22,31] (n = 1,580), the median mortality at 30 or 28 days in the control group and the HFOV group was 41.1% (range 28.6 to 52.1%) and 40.4% (range 37.3 to 43.2%), respectively. The results of the meta-analysis suggested that HFOV did not significantly reduce mortality at 30 or 28 days in adult ARDS patients (relative risk (RR) 1.051, 95% CI 0.813, 1.358; Table 2 and Figure 2A). As significant heterogeneity was detected among the above five enrolled studies (*I*^2^ = 63.1%, *P* = 0.028), and considering the tidal volume and plateau airway pressure might be the sources of heterogeneity, we performed subgroup analysis stratified by the tidal volume (≤8 ml/kg predicted body weight) and by the plateau airway pressure (≤35 cmH2O) in the control group, respectively. The results of these subgroup analyses also suggest that HFOV failed to reduce mortality at 30 or 28 days in adult ARDS patients. These results of subgroup analysis and the related forest plot are shown in Table 2 and Figure 2A, B.

**Table 2:** Main results of meta-analysis of mortality

	**Studies included (n)**	**Case number (n)**		**Heterogeneity**		**Pooled RR (95% CI)**	***P***
		**HFOV**	**CMV**	***I***^**2**^**(%)**	***P***		
**Mortality at 28 or 30 days**	5	800	780	63.1	0.028	1.051	0.704
						(0.813, 1.358)	
Subgroup							
Tidal volume in control group <8 ml/kg							
Mandated	3	688	683	63.1	0.067	1.149	0.329
						(0.869, 1.519)	
Not mandated	2	112	97	56.4	0.13	0.899	0.712
						(0.511, 1.582)	
Plateau pressure in control group <35cmH_2_O							
Mandated	2	290	286	14.6	0.279	1.323	0.092
						(0.955, 1.834)	
Not mandated	3	510	494	44.7	0.164	0.942	0.665
						(0.720, -1.233)	
**ICU mortality**	3	686	685	63.3	0.066	1.218	0.160
						(0.925, 1.604)	

CMV, conventional mechanical ventilation; HFOV, high-frequency oscillatory ventilation; n, number; RR, relative risk.

Sensitivity analysis was also analyzed, and the results demonstrated that after each study was excluded from the overall meta-analysis, similar results were obtained (Table 3). No publication bias of the enrolled studies was observed (Table 4).

**Table 3:** Sensitivity analysis

**Excluded study**	**Relative risk**	**95% CI**
**Mortality at 28 or 30 days**		
Derdak (2002)	1.164289	0.9238705, 1.4672717
Bollen (2005)	1.0212231	0.76526242, 1.3627961
Ferguson (2013)	0.94643247	0.77333957, 1.158268
Young (2013)	1.0512564	0.69253296, 1.5957943
Shah (2004)	1.0661671	0.80484247, 1.4123416
**ICU mortality**		
Demory (2007)	1.2246462	0.89132315, 1.6826202
Ferguson (2013)	1.0530359	0.89893317, 1.2335562
Young (2013)	1.4420993	1.160399, 1.7921858
**Oxygenation failure**		
Derdak (2002)	0.58428353	0.28815368, 1.1847403
Bollen (2005)	0.51638663	0.31883517, 0.83634168
Ferguson (2013)	0.82795441	0.31200156, 2.1971316
**Ventilation failure**		
Derdak (2002)	1.0543551	0.4344992, 2.5584965
Bollen (2005)	0.91356009	0.43406373, 1.9227407
Ferguson (2013)	0.64884853	0.2125513, 1.9807193
**Duration of mechanical ventilation**		
Derdak (2002)	0.09791627	−0.09584169, 0.29167423
Bollen (2005)	0.06240474	−0.06527908, 0.19008856
Young (2013)	0.15380768	−0.11983663, 0.427452
**Barotrauma**		
Shah (2004)	1.229754	0.84895152, 1.7813679
Derdak (2002)	1.3130077	0.87979519, 1.9595348
Bollen (2005)	1.1886587	0.78003234, 1.8113472
Ferguson (2013)	0.6947636	0.29700547, 1.6252108
**Hypotension**		
Derdak (2002)	2.6042271	0.44842839, 15.123928
Bollen (2005)	0.69353223	0.05379397, 8.9412804
Shah (2004)	0.89276189	0.0725222, 10.990066

**Table 4:** Publication bias

**Type of meta-analysis**	**Begg’s test**		**Egger’s test**	
	***z***	**Pr > |z|**	***t***	***P*** **> |t|**
Mortality at 28 or 30 days	0.000	1.000	−0.230	0.833
ICU mortality	0.520	0.602	0.250	0.841
Oxygenation failure	1.57	0.117	2.39	0.252
Ventilation failure	−0.52	0.602	−0.73	0.597
Duration of mechanical ventilation	1.04	0.296	1.49	0.376
Barotrauma	−0.68	0.497	−2.67	0.116
Hypotension	−0.52	0.602	−0.62	0.646

### ICU mortality

Three eligible studies [20-22] (n = 1,371) reported ICU mortality. The median mortality in the control group and the HFOV group was 30.8% (range 26.7 to 42.1%) and 44.2% (range 30.8 to 44.7%), respectively. We performed a meta-analysis using a random-effects model, and the results of this meta-analysis suggested that high-frequency oscillatory ventilation did not significantly reduce or increase the risk of death in ICU compared with conventional mechanical ventilation in adult ARDS patients (RR 1.218, 95% CI 0.925, 1.604; Table 2 and Figure 2C). However, the sensitivity analysis showed that a paradoxical result obtained after the OSCAR trial [22] was excluded from the overall meta-analysis, and it suggested that HFOV would significantly increase the ICU mortality in adult ARDS patients (RR 1.442, 95% CI 1.160, 1.792; Table 3). Neither Egger’s test nor Begg’s test showed any evidence of publication bias (Table 4).

### Oxygenation failure, ventilation failure and duration of mechanical ventilation

Oxygenation failure was defined as persisting abnormal low oxygenation index or refractory hypoxemia after the treatment of mechanical ventilation. The meta-analysis of three eligible studies [18,19,21] (n = 757) that reported the incidence of oxygenation failure demonstrated HFOV significantly reduced the risk of oxygenation failure compared with conventional ventilation (RR 0.557, 95% CI 0.351, 0.884; Figure 2D). The result of sensitivity analysis showed HFOV would not significantly improve oxygenation compared with CMV, when the study of Derdak [18] or the OSCILLATE trial [21] was excluded from the overall meta-analysis (Table 3). It suggested that the result of this meta-analysis for oxygenation failure was not stable, and that further clinical trials are needed to determine whether HFOV is more effective than CMV for the improvement of oxygenation in adult ARDS patients. No publication bias of the enrolled studies was observed (Table 4).

Three enrolled studies demonstrated the ventilation efficiency of HFOV: one study [21] reported the incidence of refractory acidosis, one [19] reported the occurrence of acidosis, and the third [18] reported the incidence of ventilation failure with a clear definition, that is, ‘a pH ≤ 7.15 for 6 hours and a bicarbonate of 19 meq/L or more’. We performed a meta-analysis with a random-effects model and demonstrated that there was no significant difference in ventilation efficiency between the HFOV group and the control group (RR 0.892, 95% CI 0.435, 1.829; Figure 2E). Sensitivity analysis demonstrated that after each study was excluded from the overall meta-analysis, similar results were obtained (Table 3). No publication bias of the enrolled studies was observed (Table 4).

Three eligible trials [18,19,22] (n = 1,004) provided the duration of mechanical ventilation. The results of meta-analysis showed that the high-frequency oscillation strategy did not significantly reduce the duration of mechanical ventilation (standardized mean difference 0.079, 95% CI −0.045, 0.203; Figure 2F). Sensitivity analysis demonstrated that after each study was excluded from the overall meta-analysis, similar results were obtained (Table 3). No publication bias of the enrolled studies was observed (Table 4).

### Adverse event: barotrauma

Barotrauma was defined as a group of symptoms caused by the high airway pressure during mechanical ventilation, such as pneumothorax, pneumomediastinum, pneumopericardium, subcutaneous emphysema and so on. We performed a meta-analysis to summarize the difference in the risk of barotrauma between the HFOV group and control group. Four enrolled studies provided the incidence of barotrauma, but they all applied different definitions of barotrauma: only pneumothorax [31], any pulmonary air leak [18], severe air leak resulting in treatment failure [19], or new-onset barotrauma [21]. The above four enrolled trails reported the incidence of barotrauma in the HFOV group as 0/15 [31], 7/75 [18], 1/37 [19], 46/256 [21] patients, respectively; and in the CMV group as 1/13 [31], 9/73 [18], 1/24 [19], 34/259 [21] patients, respectively. The results of the meta-analysis and the forest plot are shown in Figure 2G. The relative risk for barotrauma was 1.205 (95% CI 0.834, 1.742). This result suggests that HFOV does not increase or reduce the risk of barotrauma compared with CMV. In the sensitivity analysis, similar results were obtained after each study was excluded from the overall meta-analysis (Table 3). No publication bias of the enrolled studies was observed (Table 4).

### Adverse event: hypotension

Three eligible trials (n = 237) reported the incidence of hypotension in the HFOV group as 0/75 [18], 4/37 [19], 1/15 [31] patients, respectively; and in the CMV group as 2/73 [18], 1/24 [19], 0/13 [31] patients, respectively. The results of meta-analysis demonstrated that the HFOV would not significantly increase the risk of hypotension compared with CMV (RR 1.326, 95% CI 0.271, 6.476; Figure 2H). Sensitivity analysis demonstrated that after each study was excluded from the overall meta-analysis, similar results were obtained (Table 3). No publication bias of the enrolled studies was observed (Table 4).

## Discussion

To the best of our knowledge, the present study is the first meta-analysis examining the effect of HFOV in unique adults with ARDS, although two cognate Cochrane reviews [27,32] have been published based on studies including mutually exclusive groups of patients, and the more recent one [32] combined the results from adult and pediatric patients. Importantly, the efficacy of HFOV is likely associated with the age of the patient, as Arnold *et al*. reported that patients older than five years had dramatically increased mortality compared with patients younger than five years, when treated with HFOV [33]. Additionally, the earlier Cochrane review [27] found only two small RCTs and was not powerful enough to draw definitive conclusions. Although the later one [32] included eight randomized controlled trials with 419 patients, it included two studies investigating the combination effect of HFOV and additional interventions (prone positioning [34] and tracheal gas insufflation [35]), which would complicate the results of meta-analysis.

In the present meta-analysis of mortality, we showed that HFOV did not significantly reduce mortality at 30 or 28 days compared with CMV. This finding contrasts sharply with experimental studies in animals in which benefits of high-frequency oscillation were observed [36]. Our results may suggest that the benefits of HFOV cannot be translated directly from animal models to adult ARDS patients, probably because there is great heterogeneity in the recruitability of the lung [37] and because the well-controlled conditions of animal studies are often difficult to replicate in human clinical trials. Our results are also at variance with those of the latest Cochrane review of HFOV in 2013 [32], which showed HFOV significantly reduced in-hospital or 30-day mortality compared with conventional ventilation. This may be simply because the present meta-analysis enrolled two more large multicenter trials and recruited more than three times the number of patients recruited in the previous meta-analysis. Our results are consistent with those of two recently published large-scale RCTs, which are known as the OSCILLATE trial [21] and OSCAR trial [22], respectively. Sensitivity analysis showed that in the meta-analysis of mortality at 30 or 28 days, after each study was excluded from the overall meta-analysis, similar results were obtained. This suggests that our results of 30- or 28-day mortality are valid.

As moderate heterogeneity was revealed in the overall meta-analysis of mortality, and considering that the tidal volume and plateau airway pressure might be the sources of heterogeneity, we performed subgroup analysis according to the tidal volume (≤8 ml/kg predicted body weight) and according to the plateau airway pressure (≤35 cmH2O) in the control group, respectively. Although the heterogeneity remained moderate after the stratification by tidal volume, the heterogeneity was significantly reduced when the data were stratified depending on the control group’s plateau airway pressure. It appears that the plateau airway pressure is one of the most possible sources of heterogeneity. The results from these subgroup analyses all suggest that HFOV did not significantly reduce mortality at 30 or 28 days in adult ARDS patients. This indicates that the results of this meta-analysis are stable.

Although the meta-analysis of ICU mortality demonstrated that HFOV did not significantly affect ICU mortality compared with CMV, the sensitivity analysis showed that the OSCAR trial [22] drastically affected the pooled results of our meta-analysis of ICU mortality. After the OSCAR trial was excluded, the pooled results suggested that the application of HFOV would significantly increase ICU mortality compared with CMV. Additionally, the beneficial effect of HFOV on mortality could have been underestimated because one study [21] enrolled 40% of the patients included in our meta-analysis of ICU mortality, and in that study more than 10% of patients in the control group crossed over to receive HFOV. Thus, the result of this meta-analysis of the risk of death in ICU was not stable, and further clinical trials are needed to determine whether HFOV is as effective as CMV for the improvement of ICU survival in adult ARDS patients.

The present meta-analysis showed that HFOV significantly reduced the risk of hypoxemia compared with CMV. This result is consistent with our meta-analysis of the PaO_2_/FiO_2_ ratio on day 1 which demonstrated that the application of HFOV significantly improved the PaO_2_/FiO_2_ ratio on the first day after the initiation of mechanical ventilation (data not shown). However, the sensitivity analysis showed whenever the OSCILLATE trial [21] or the study by Derdak *et al*. [18] was excluded, the pooled results of the other two studies suggested that HFOV did not significantly reduce the incidence of oxygenation failure. This may be simply because the other two trials enrolled too few patients to identify a significant difference. Thus, it is still possible that the application of HFOV significantly improves oxygenation and reduces the risk of hypoxemia.

There are several limitations of the present meta-analysis. First, the six enrolled studies were published between 2002 and 2013, which may have caused moderate heterogeneity of the control group and therefore complicated the results of this meta-analysis. Second, the sample size of the included trials ranged from 28 to 795, which could have influenced the precision of the pooled effect-estimates. Lastly, we only evaluated the effect of HFOV on the incidence of barotrauma and hypotension, therefore, the evaluation of the safety of HFOV might not be comprehensive enough.

## Conclusions

The pooled analysis of the currently available data suggests that HFOV does not significantly reduce mortality at 30 or 28 days, nor does it reduce ICU mortality, although it seems to significantly reduce the risk of oxygenation failure in adult ARDS patients. Furthermore, HFOV has no significant effect on the incidence of ventilation failure, the duration of mechanical ventilation or the risk of barotrauma and hypotension. Although these results suggest HFOV would not increase the incidence of barotrauma or hypotension, they do not support the recommendation that HFOV is used in routine care for adult patients with ARDS. More large-scale multicenter RCTs are required to further determine the efficacy and safety of HFOV in adult ARDS patients.

## Key messages

•In adult ARDS patients, high-frequency oscillation did not significantly reduce mortality at 30 or 28 days, or the mortality within the ICU compared with CMV.

•Compared with conventional ventilation, HFOV, although having no significant effect on the incidence of ventilation failure or the duration of mechanical ventilation, significantly reduced the risk of oxygenation failure.

•High-frequency oscillatory ventilation was not associated with an increased risk of barotrauma or hypotension and seemed to be as safe as conventional ventilation.

## Abbreviations

ALI: acute lung injury; ARDS: acute respiratory distress syndrome; CMV: conventional mechanical ventilation; FiO_2_: fraction of inspired oxygen; HFOV: high-frequency oscillatory ventilation; PaO_2_: arterial oxygen tension; PEEP: positive end expiratory pressure; RCT: randomized controlled trial; RR: relative risk; SMD: standardized mean difference.

## Competing interests

The authors declare that they have no competing interests.

## Authors’ contributions

All authors conceived the study and contributed to the study design. XLG and GNW performed the literature review and data extraction. YWY and DHS performed statistical analysis. GNW, DHS and YS analyzed and interpreted the data. XLG, DHS and YWY drafted the manuscript. XLG, GNW and YS were responsible for the revision of the manuscript for important intellectual content. YS supervised the study. All authors have read and approved the manuscript for submission.

## Supplementary Material

Additional file 1 **The names of all ethical bodies involved in the six enrolled trials.** All ethical body names involved in the six trials that were enrolled in the present meta-analysis have been listed in this file. Click here for file
