# Supplementary material for: Is high-frequency oscillatory ventilation more effective and safer than conventional protective ventilation in adult acute respiratory distress syndrome patients? A meta-analysis of randomized controlled trials
Source: Crit Care. 2014 May 30;18(3):R111. doi: 10.1186/cc13900 (PMC4095578; doi:10.1186/cc13900)
Supplement: Additional file 1 — The names of all ethical bodies involved in the six enrolled trials. All ethical body names involved in the six trials that were enrolled in the present meta-analysis have been listed in this file. [file cc13900-S1.pdf]

Table. S1 The names of all ethical bodies involved in the six enrolled trials

First author	Year	Names of ethical bodies	
Derdak[18]	2002	Ethical committee board of	Participating institutions as follow:
			Wilford Hall Medical Center/Brooke Army Medical Center
			Mt. Sinai Hospital/Wellesley Hospital, University of Toronto
			Barnes Jewish Hospital
			University Health Network, University of Toronto
			University of Virginia Medical Center
			Maine Medical Center
			Loma Linda University Medical Center
			Sunnybrook and Women's College Health Sciences Center, University of Toronto
			Allegheny General Hospital
			Bronson Methodist Hospital
Bollen[19]	2005	Ethical committee board of	Participating institutions as follow:
			St Thomas Hospital, London
			University Hosp ital of Wales, Cardiff
			Hopital Cochin, Paris
			University Hospital Mainz, Germany
			University Medical Centre Utrecht, The Netherlands
Demory[20]	2007	Ethical committee board of	Participating institution as follow:
			Comité Consultatif de Protection des Personnes dans la Recherche Biomédicale de Marseille
Ferguson[21]	2013	Ethical committee board of	Participating institutions as follow:
			Toronto General Hospital
			Toronto Western Hospital
			Mount Sinai Hospital
			St. Michael's Hospital
			Sunnybrook Health Science Centre
			St. Joseph's Hospital

			Hamilton Health Sciences
			St. Joseph's Healthcare
			University of Western Ontario
			Royal Victoria Hospital
			Ottawa Hospital General Campus
			Ottawa Hospital Civic Campus
			Hôpital Maisonneuve - Rosemont
			Centre Hospitalier del' Universite de Montreal Saint Luc
			Hôpital du Sacré - Coeur de Montreal
			Centre Hospitalier de l' Universite de Sherbrooke
			Hôpital L' Enfant Jesus
			Centre de Santé et de Services Sociaux de St. Jérôme
			Queen Elizabeth II Health Sciences Centre
			Vancouver Island Health Authority
			Vancouver General Hospital
			St. Paul's Hospital
			Royal Columbian Hospital
			Surrey Memorial Hospital
			University of Alberta
			Health Sciences Centre
			Scott & White Memorial Hospital
			University of Texas Health Science Centre
			Parkland Memorial Hospital
			John Peter Smith Hospital
			Orlando Regional Medical Centre
			University of Michigan Ann Arbor
			Denver Health Medical Centre
			Brody School of Medicine at East Carolina University
			King Saud Bin Abdulaziz University for Health Science
			Riyadh Armed Forces Hospital
			Pontifica Universidad Catolica de Chile
			Deenanath Mangeshkar Hospital & Research Centre
Young[22]	2013	Ethical committee board of	Participating institutions as follow:
			John Radcliffe Hospital, Oxford
			Derriford Hospital, Plymouth
			Aberdeen Royal Infirmary, Aberdeen

			Medway Hospital, Gillingham
			Queen Elizabeth Hospital, Birmingham
			Royal Sussex County Hospital, Brighton
			University College Hospital, London
			University Hospital of Wales, Cardiff
			Royal United Hospital, Bath
			Manchester Royal Infirmary, Manchester
			Ysbyty Maelor Hospital, Wrexham
			Queen Elizabeth Hospital, Gateshead
			Stirling Royal Infirmary, Stirling
			Royal Cornwall Hospi tal, Truro
			Wythenshawe Hospital, Manchester
			University Hospital of North Staffordshire , Stoke- on-Trent
			Ipswich Hospital, Ipswich
			Manchester Royal Infirmary (Cardiac), Manchester
			James Paget Hospital, Great Yarmouth
			Queen Alexandra Hospital, Portsmouth
			Queen Margaret Hospital, Dunfermline
			Royal Blackburn Hospital, Blackburn
			Leeds General Infirmary, Leeds
			Southampton General Hospital, Southampton
			St James University Hospital, Leeds
			York Hospital, York
			Victoria Hospital, Blackpool
			Southend Hospital, Westcliff- on- Sea
			Royal Victoria Infirmary, Newcastle
Shah[30]	2004	Ethical committee board of	Participating institution as follow
			University Hospital of Wales, Cardiff, United Kingdom
